# Efficacy of sub lethal concentration of entomopathogenic fungi on the feeding and reproduction of *Spodoptera litura*

**DOI:** 10.1186/s40064-015-1437-1

**Published:** 2015-11-06

**Authors:** P. Vinayaga Moorthi, C. Balasubramanian, S. Selvarani, A. Radha

**Affiliations:** PG and Research Department of Zoology, Thiagarajar College (Autonomous), Madurai, 625 009 Tamil Nadu India; Kunthavai Nacchiyar Government Arts College for Women (Autonomous), Thanjavur, India

**Keywords:** *Spodoptera litura*, *Isaria fumosorosea*, *Paecilomyces variotii*, *Beauveria bassiana*, Growth rate, Secondary metabolite

## Abstract

In the present investigation, impact of sub lethal concentrations of entomopathogenic fungi, namely *Isaria fumosorosea*, *Beauveria bassiana* and *Paecilomyces variotii*, secondary metabolite on feeding, growth, fecundity and hatchability of *Spodoptera litura* was performed. The *S. litura* treated with *I. fumosorosea* and *B. bassiana* metabolites exhibited renounced food consumption. The growth rate of treated *S. litura* with metabolite of *I. fumosorosea* had drastic reduction. In the case of approximate digestibility (AD), maximum impact was established by the *I. fumosorosea* isolate, which significantly reduced the approximate digestibility of the IV and V instar larvae. The III instar larvae of *S. litura* treated with *I. fumosorosea* metabolite showed significantly lower efficiency of conversion of digested food (ECD) and efficiency of conversion of ingested food (ECI) values than IV and V instars. However the performance of metabolites on fecundity and hatchability of *S. litura* was immense. Therefore, metabolites of *I. fumosorosea* could be reliable biocontrol agent, which has been highly recommended for *S. litura* management in commercial crops.

## Background

Entomopathogenic fungi, a group of microbial pest control agents, are natural insect pathogens regulating the insect population in an environment. It has been widely employed for the control of major insect pest and currently 700 species have been reported as entomopathogenic (Suganya and Selvanarayanan 2010). There are several insect pathogens, like *Beauveria bassiana, Metarhizium anisopliae, Isaria fumosorosea, Verticillium lecanii* and *Nomuraea rileyi* have been found to be promising in the control of several agricultural insect pests (Lingappa et al. 2005). The invasion of these fungi, during pathogenesis has been facilitated by enzymes but it has been strongly defended by the insect’s cellular and humoral reaction. In climax, the insect barrier has been perfectly broken by the fungi by its enzymes and they started growing inside the hemolymph, where toxins like oosporein, beauverin and destruxin were secreted. The studies on role of enzyme in pathogenicity was studied enormously, while the release of secondary soluble fungal toxin during post penetration event has been detailed very little (Ortiz-Urquiza et al. 2010). Robert (1981) provided a complete overview on these fungal toxins. According to Wang et al. (2007) studies, it has been observed that, the fungal metabolites are potential insecticide against insect pest. Thomsen and Eilenberg (2000) stated that the lepidopteran insects are vulnerable to the destruxins. In support of this, Hu et al. (2007) stated that, the secondary metabolites produced by the entomopathogenic fungi particularly *M. anisopliae,* were toxic to *Spodoptera litura.* Therefore, the present study, aims to study the role of secondary metabolite of entomopathogenic fungi on the feeding, growth and development of *S. litura.*

## Results

### Isolation of entomopathogenic fungi

Entomopathogenic fungi were isolated from the rhizosphere soil collected from Kalloorani, (N9°28.575′ E78°09.96) Virudhunagar District, Tamil Nadu, India. It was identified as *Isaria fumosorosea*, *Beauveria bassiana* (JX481967) and *Paecilomyces variotii* (JX481968) based on the Internal Transcript Spacer (ITS) sequencing.

### Screening of secondary metabolites

The selected entomopathogenic fungi were subjected to solvent extraction (Ethyl acetate) for the isolation of active secondary metabolites. The extracts of the entomopathogenic fungi were subjected to study the Rf value, by separating them by using TLC and were observed under UV trans-illuminator and ninhydrin spray.

### Feeding experiment

Different sub lethal concentrations were used for the feeding experiment study against *S. litura* in in vitro. The feeding experiment was performed on different parameters such as consumption index (CI), growth rate (GR), approximate digestibility (AD), conversion of digested food (ECD) and conversion of Ingested food (ECI) (Tables 1, 2, 3). The one tailed t test analysis report was presented in Table 4.

**Table 1:** Effect of ethyl acetate fraction of *Isaria fumosorosea* on the growth rate, consumption index and approximate digestibility of 3rd, 4th and 5th instar larvae of *Spodoptera litura*

*I. fumosorosea fraction* (ppm)	CI	GR	AD	ECD	ECI
3rd					
C	0.892 ± 0.002^a^	0.193 ± 0.001^a^	99.09 ± 0.047^a^	23.80 ± 0.057^a^	22.30 ± 0.085^b^
1	0.310 ± 0.017^b^	0.189 ± 0.001^c^	97.93 ± 0.028^b^	23.01 ± 0.077^b^	23.07 ± 0.069^a^
2	0.226 ± 0.003^e^	0.185 ± 0.001^d^	97.16 ± 0.023^c^	18.44 ± 0.068^c^	17.92 ± 0.024^c^
3	0.251 ± 0.003^d^	0.183 ± 0.001^b^	96.92 ± 0.002^d^	17.89 ± 0.029^d^	17.52 ± 0.026^d^
4	0.276 ± 0.001^c^	0.180 ± 0.0004^a^	96.92 ± 0.030^d^	11.00 ± 0.081^c^	10.92 ± 0.012^e^
CD at 0.01	0.006	0.003	0.134	0.067	0.044
CD at 0.05	0.004	0.002	0.092	0.046	0.030
4th					
C	0.926 ± 0.001^a^	0.198 ± 0.0008^a^	98.71 ± 0.123^a^	96.52 ± 0.098^a^	99.23 ± 0.183^a^
1	0.147 ± 0.001^e^	0.197 ± 0.0008^b^	95.86 ± 0.050^b^	95.46 ± 0.098^a^	99.14 ± 0.446^a^
2	0.280 ± 0.012^b^	0.192 ± 0.0008^c^	91.12 ± 0.138^c^	95.34 ± 0.014^a^	99.04^a^ ± 0.438^b^
3	0.247 ± 0.001^c^	0.187 ± 0.0008^c^	90.32 ± 0.106^d^	94.36 ± 0.094^a^	98.91 ± 0.116^b^
4	0.234 ± 0.002^d^	0.185 ± 0.001^d^	89.52 ± 0.077^e^	80.00 ± 0.172^b^	74.17 ± 0.130^c^
CD at 0.01	0.003	0.005	0.126	1.79	1.334
CD at 0.05	0.002	0.002	0.087	1.27	0.917
5th					
C	1.112 ± 0.003^a^	0.201 ± 0.0008^a^	97.34 ± 0.046^a^	97.92 ± 0.069^d^	99.54 ± 0.087^a^
1	1.077 ± 0.020^b^	0.195 ± 0.001^b^	91.34 ± 0.046^b^	96.72 ± 0.111^b^	99.53 ± 0.033^a^
2	1.060 ± 0.020^c^	0.189 ± 0.001^c^	90.32 ± 0.053^c^	96.24 ± 0.086^c^	99.25 ± 0.082^b^
3	0.867 ± 0.002^d^	0.187 ± 0.001^c^	89.17 ± 0.058^d^	96.47 ± 0.077^a^	99.17 ± 0.045^c^
4	0.838 ± 0.003^e^	0.181 ± 0.001^c^	88.05 ± 0.061^e^	96.87 ± 0.048^e^	99.03 ± 0.073^d^
CD at 0.01	0.001	0.004	0.023	0.050	0.147
CD at 0.05	0.001	0.003	0.016	0.035	0.105

*CI* consumption index, *GR* growth rate, *AD* approximate digestibility, *ECD* efficiency of conversion of digested food, *ECI* efficiency of conversion of ingested food

^a,b,c,d^ *CD* critical difference values were significant at 0.01 and 0.05 % level

**Table 2:** Effect of fraction of *Paecilomyces variotii* on the growth rate, consumption index and approximate digestibility 3rd, 4th and 5th instar larvae of *Spodoptera litura*

*P. variotii fraction* (ppm)	CI	GR	AD	ECD	ECI
3rd					
C	0.867 ± 0.003^a^	0.200 ± 0.001^a^	96.92 ± 0.038^a^	94.27 ± 0.014^a^	98.11 ± 0.04^a^
1	0.694 ± 0.011^b^	0.196 ± 0.001^b^	89.47 ± 0.089^b^	93.25 ± 0.29^b^	97.61 ± 0.11^b^
2	0.615 ± 0.61^c^	0.194 ± 0.002^c^	88.67 ± 0.036^b^	92.46 ± 0.18^d^	97.36 ± 0.17^c^
3	0.580 ± 0.012^d^	0.194 ± 0.001^c^	85.71 ± 0.084^b^	92.87 ± 0.17^c^	97.22 ± 0.34^d^
4	0.471 ± 0.003^e^	0.193 ± 0.002^c^	83.33 ± 0.019^c^	23.01 ± 0.39^e^	22.30 ± 0.06^e^
CD at 0.01	0.006	0.003	2.397	0.043	0.042
CD at 0.05	0.045	0.002	1.648	0.030	0.029
4th					
C	0.926 ± 0.002^a^	0.207 ± 0.000^a^	92.71 ± 0.123^a^	91.32 ± 0.026^a^	99.54 ± 0.098^a^
1	0.227 ± 0.000^b^	0.198 ± 0.001^b^	91.33 ± 0.026^d^	89.54 ± 0.086^b^	99.54 ± 0.192^a^
2	0.190 ± 0.003^c^	0.192 ± 0.001^c^	89.74 ± 0.070^b^	89.10 ± 0.070^b^	99.43 ± 0.184^ab^
3	0.140 ± 0.004^e^	0.191 ± 0.000^d^	89.33 ± 0.026^c^	87.64 ± 0.026^c^	99.36 ± 0.061^a^
4	0.161 ± 0.001^d^	0.187 ± 0.000^e^	87.64 ± 0.049^e^	80.50 ± 0.102^d^	74.17 ± 0.050^c^
CD at 0.01	0.003	0.008	1.533	0.035	0.162
CD at 0.05	0.002	0.006	1.054	0.024	0.111
5th					
C	1.006 ± 0.002^a^	0.215 ± 0.000^a^	94.62 ± 0.066^a^	96.47 ± 0.104^a^	99.46 ± 0.053^a^
1	0.241 ± 0.001^b^	0.192 ± 0.001^b^	93.07 ± 0.041^b^	96.37 ± 0.184^b^	99.09 ± 0.024^c^
2	0.115 ± 0.001^d^	0.190 ± 0.002^c^	92.68 ± 0.085^c^	95.47 ± 0.086^c^	99.38 ± 0.046^b^
3	0.107 ± 0.002^e^	0.187 ± 0.000^d^	92.02 ± 0.390^d^	95.46 ± 0.140^c^	99.03 ± 0.045^d^
4	0.139 ± 0.001^c^	0.175 ± 0.000^e^	91.34 ± 0.057^e^	94.36 ± 0.180^d^	98.78 ± 0.037^e^
CD at 0.01	0.003	0.008	0.036	0.117	0.120
CD at 0.05	0.002	0.006	0.025	0.080	0.083

*CI* consumption index, *GR* growth rate, *AD* approximate digestibility, *ECD* efficiency of conversion of digested food, *ECI* efficiency of conversion of ingested food

^a,b,c,d^ *CD* critical difference values were significant at 0.01 and 0.05 % level

**Table 3:** Effect of ethyl acetate fraction of *Beauveria bassiana* on the growth rate, consumption index and approximate digestibility of 3rd, 4th and 5th instar larvae of *Spodoptera litura*

*B. bassiana fraction* (ppm)	CI	GR	AD	ECD	ECI
3rd					
C	1.94 ± 0.002^a^	0.199 ± 0.003^ab^	99.01 ± 0.085^a^	23.01 ± 0.100^a^	22.90 ± 0.065^b^
1	1.77 ± 0.001^b^	0.195 ± 0.000^c^	97.93 ± 0.057^b^	22.93 ± 0.156^a^	98.00 ± 0.054^a^
2	1.42 ± 0.001^c^	0.191 ± 0.001^b^	96.92 ± 0.041^c^	14.5 ± 0.143^b^	14.70 ± 0.037^c^
3	1.36 ± 0.003^d^	0.189 ± 0.001^ab^	96.26 ± 0.150^d^	14.8 ± 0.132^b^	14.70 ± 0.022^c^
4	0.867 ± 0.002^c^	0.176 ± 0.000^c^	92.45 ± 0.040^e^	12.24 ± 0.190^c^	11.33 ± 0.066^d^
CD at 0.01	0.009	0.003	0.089	0.324	0.310
CD at 0.05	0.006	0.002	0.061	0.223	0.213
4th					
C	1.017 ± 0.002^a^	0.201 ± 0.002^c^	96.92 ± 0.061^a^	83.11 ± 0.123^a^	81.90 ± 0.046^a^
1	0.624 ± 0.000^b^	0.192 ± 0.000^b^	96.11 ± 0.021^b^	69.00 ± 0.141^a^	64.18 ± 0.047^a^
2	0.310 ± 0.000^e^	0.189 ± 0.001^ab^	93.45 ± 0.034^d^	68.22 ± 0.138^a^	62.93 ± 0.047^b^
3	0.317 ± 0.003^d^	0.187 ± 0.000^a^	89.24 ± 0.032^e^	58.11 ± 0.147^b^	55.28 ± 0.094^c^
4	0.361 ± 0.001^c^	0.171 ± 0.001^b^	87.12 ± 0.078^c^	33.33 ± 0.132^c^	32.00 ± 0.074^d^
CD at 0.01	0.001	0.005	0.002	0.130	0.002
CD at 0.05	0.001	0.003	0.001	0.089	0.002
5th					
C	1.091 ± 0.002^a^	0.202 ± 0.002^a^	96.92 ± 0.047^a^	98.42 ± 0.111^a^	93.24 ± 0.047^b^
1	0.297 ± 0.000^b^	0.191 ± 0.001^b^	88.80 ± 0.047^b^	94.36 ± 0.145^c^	83.0 ± 0.0081^c^
2	0.229 ± 0.002^c^	0.190 ± 0.001^b^	87.75 ± 0.081^c^	76.36 ± 0.156^b^	71.26 ± 0.009^a^
3	0.183 ± 0.002^e^	0.186 ± 0.002^ab^	86.33 ± 0.094^d^	54.36 ± 0.141_b_	49.23 ± 0.081^a^
4	0.217 ± 0.006^d^	0.169 ± 0.002^c^	86.33 ± 0.094^d^	23.01 ± 0.127^d^	22.90 ± 0.047^d^
CD at 0.01	0.002	0.003	0.039	0.958	1.974
CD at 0.05	0.001	0.001	0.027	0.657	1.357

*CI* consumption index, *GR* growth rate, *AD* approximate digestibility, *ECD* efficiency of conversion of digested food, *ECI* efficiency of conversion of ingested food

^a,b,c,d^ *CD* critical difference values were significant at 0.01 and 0.05 % level

**Table 4:** One tailed t test analysis for control and treatment groups of the CI, GR, AD, ECD and ECI of instars of *Spodoptera litura* treated with Entomopathogenic fungal fraction

	Consumption index			Growth rate			Approximate digestibility			Efficiency of conversion of digested food			Efficiency of conversion of ingested food		
	III	IV	V	III	IV	V	III	IV	V	III	IV	V	III	IV	V
*Isaria fumosorosea*															
Df	3	3	3	3	3	3	3	3	3	3	3	3	3	3	3
t Stat	2.46	25.06	33.84	3.18	2.71	4.25	5.960	4.861	9.412	2.15	1.41	10.34	2.37	1.04	2.51
P(T ≤ t) one-tail	0.04*	6 × 10^−5^**	2 × 10^−5^**	0.02*	0.03*	0.01*	0.004**	0.008**	0.001**	0.06*	0.12	0.0009**	0.048*	0.18^ns^	0.04*
*Paecilomyces variotii*															
Df	3	3	3	3	3	3	3	3	3	3	3	3	3	3	3
t Stat	5.96	38.05	26.91	5.96	6.58	9.79	6.86	4.12	1.22	1.08	2.17	2.48^ns^	1.04	1.03	2.93
P(T ≤ t) one-tail	0.004*	2 × 10^−05^**	5 × 10^−05^**	0.004**	0.003**	0.001**	0.003**	0.012*	0.15^ns^	0.17^ns^	0.05*	0.04*	0.18^ns^	0.18^ns^	0.03*
*Beauveria bassiana*															
Df	3	3	3	3	3	3	3	3	3	3	3	3	3	3	3
t Stat	3.11	8.10	33.01	2.56	3.16	3.16	2.65	2.57	15.69	2.94	3.11	2.28	0.53	3.78	2.76
P(T ≤ t) one-tail	0.026	0.001	3 × 10^−05^	0.04	0.025	0.025	0.038	0.040	0.000	0.030	0.02	0.05	0.31	0.01	0.03

*ns* not significant at 0.05 % level

* Significant at 0.05 %

** Significant at 0.01 % level

### Feeding experiment

The *S. litura* treated with *I. fumosorosea* and *B. bassiana* fractions exhibited reduced food consumption. *I. fumosorosea* fraction diminished the consumption efficiency at the mean difference of 0.035, 0.582 and 0.692 with respect to III, IV and V instar larvae of *S. litura* (Table 1) with respect from control to 1, 2, 3, 4 ppm accordingly. The calculated CD (0.05 %) (0.002, 0.002 and 0.002) was comparatively lower than that of the mean difference (0.004, 0.699 and 0.765) which implies the significant difference between control and treatment. The GR of treated *S. litura* with fractions of *I. fumosorosea* had drastic reduction in III, IV and V instar larvae ranged from 0.193 (control) to 0.180 (4 ppm) mg dry wt^−1^ live larvae^−1^; 0.198 (control) to 0.185 (4 ppm) mg dry wt^−1^ live larva^−1^ and 0.201 (control) to 0.181 (4 ppm) mg dry wt^−1^ live larva^−1^. The mean difference of the growth rate of the control and treatment was 0.004, 0.013 and 0.006, which was higher than the calculated CD value (0.05 % level) (0.002, 0.002 and 0.003). Similarly, in the *P. varioti* isolate, there was a sharp decrease in the growth rate observed (Table 2). The reduction in terms of weight was 0.07, 0.010 and 0.20 mg dry wt^−1^ live larva^−1^. The mean difference was significantly differed from control. In the case of *B. bassiana*, the reduction of growth rate was found higher in 3rd instar larvae, whereas it become remain the same in the 4th and 5th instar larvae (0.020 mg dry wt^−1^ live larva^−1^) (Table 2). The mean difference of the growth rate between the control and treatment was 0.004, 0.009 and 0.011. It was higher than the calculated CD (0.05 % level) value (0.002, 0.003 and 0.001), which implies the significant difference in the growth rate of control and experiment.

The AD sharply decreased with increasing the concentration. The maximum impact was established by the *I. fumosorosea* isolate, which significantly reduced the AD of the IV and V instar larvae. The III instar exhibited over 90 % AD which declined sharply in the IV and V instars to a maximum of 88.05 and 83.33 %. The mean difference of the control and treatment with respect to III, IV and V instar larvae was 1.16, 2.85 and 0.006. Similar reduction was not observed in *P. variotii*, while *B. bassiana* isolates exhibited the similar pattern of reduction from III to V instar larvae. The AD of the *P. variotii* and *B. bassiana* isolate from III to V instar at 4 ppm ranged from 83.33 (Table 2) to 91.34 % (Table 2) and 92.45 (Table 3) to 86.33 % (Table 3) respectively. The *P. variotii* treated V instar larvae showed healthy by maintain over 90 % AD. The CD values of the *P. variotii* and *B. bassiana* stated that, the AD of the tested instars was significantly differed from control group.

The III instar larvae of *S. litura* treated with *I. fumosorosea* toxin showed significantly lower ECD and ECI values than IV and V instars (Table 1). The similar impact was also observed in the III instar larvae (Table 2) treated with fraction of *P. variotii* (ECD and ECI) and *B. bassiana* (Table 3) (ECD and ECI). Exceptionally, V instar larvae of *S. litura* treated with *B. bassiana* had outrageous impact and have least conversion efficiency of 23.01 and 22.90 % with respect to ECD and ECI. There was a significant difference in the control and treatment was observed for ECD and ECI values of *S. litura* treated with *P. variotii* and *B. bassiana*.

### Effect of ethyl acetate fraction on the fecundity and hatchability

**Fig. 1:**
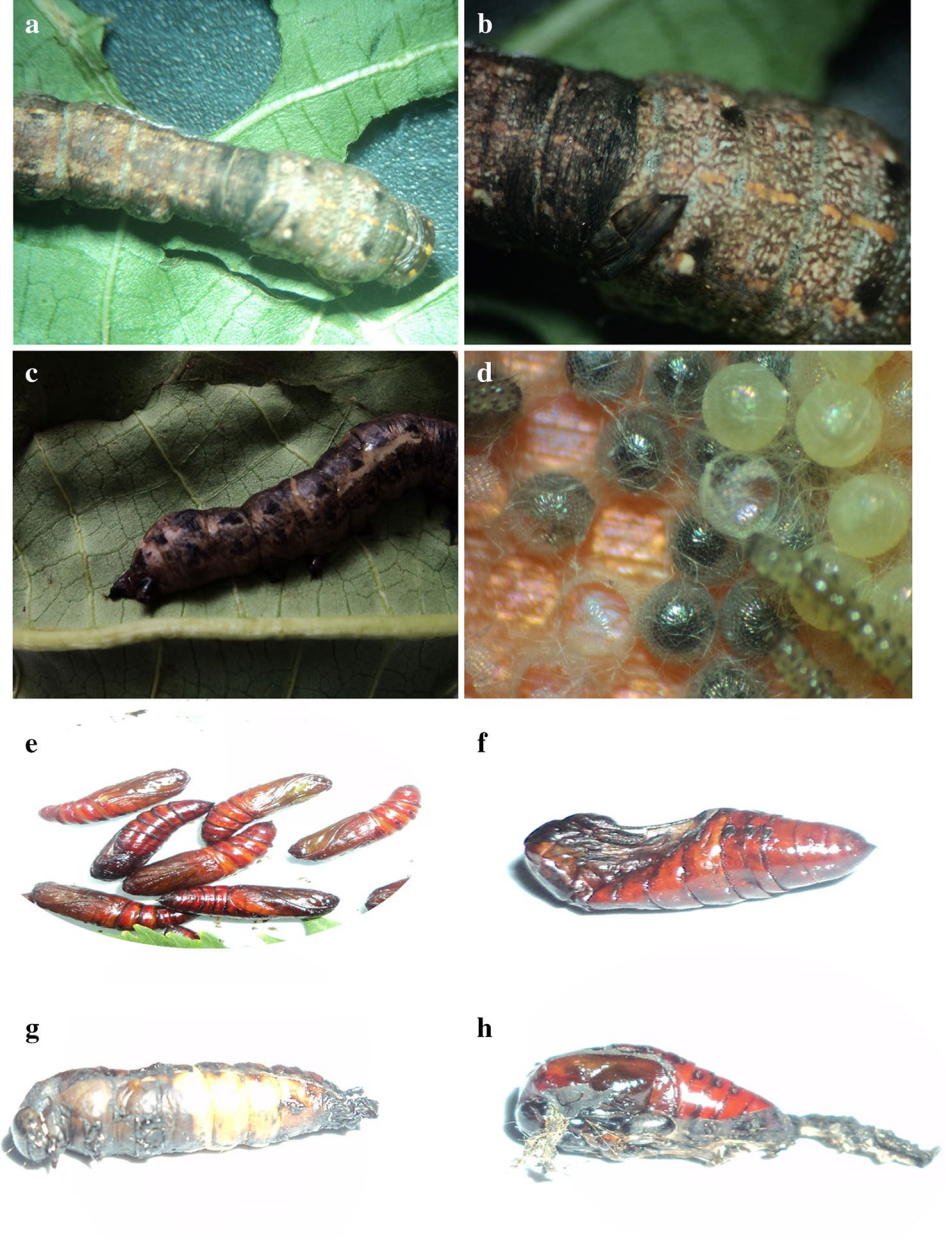
Impact sub lethal dose of secondary metabolite of *Isaria fumosorosea* on larvae and pupa and egg of *Spodoptera litura.* **a**, **b** Infected larvae and its enlarged image; **c** dead larvae; **d** unhatched eggs and hatched larvae; **e** pupa (control); **f**, **g**, **h** different type of infected pupa

The fractions of EPF had adverse effect on egg laying as well as growth stages of *S. litura*. The fecundity of *S. litura* inversely proportional to the concentration of the fraction of *I. fumosorosea* used (Table 5; Fig. 1). The fecundity of the *S. litura* treated with *I. fumosorosea* fraction drastically reduced since lowest concentrations recorded 167 eggs^−1^ batch^−1^ insect^−1^, while at 5 µl showed 67 eggs^−1^ batch^−1^ insect^−1^. The mean difference between the control and experiment was 8.07, which was higher than the calculated CD value (0.049 and 0.034 at 0.01 and 0.05 % respectively). The difference in fecundity found between the lower to higher concentration was 100 eggs batch^−1^ insect^−1^. No such reduction in the egg laying was observed in the case of *P. variotii* (Table 5) and *B. bassiana* (Table 5) treated *S. litura*. Mean while, fecundity reduction contributed by *P. variotii* and *B. bassiana* treated instars were significant at 0.01 (0.028) and 0.05 % (0.019) level. The egg laid by the *S. litura* treated with *B. bassiana* was 136 eggs bactch^−1^ insect^−1^, which was little higher than *P. variotii* treated instar. Further, an average of 55 % reduction in hatchability was observed in *I. fumosorosea*, *P. variotii* and *B. bassiana*. In comparison to the control and experimental group with calculated CD value (0.079 and 0.056 with respect to 0.01 and 0.05 %) implies the existence of significant difference in the treated group. Similarly, 76 % reduction of pupation was observed in *I. fumosorosea* fraction treated groups whereas *P. variotii* and *B. bassiana* contributed 48 and 68 % respectively. The mean difference and calculated CD at 0.01 % (3.906) and 0.05 % (2.746) showed that, the treated group was significantly differed. Similarly higher number of malformed pupae was observed in *B. bassiana* followed by *I. fumosorosea* and *P. variotii*. In this, the effect of 1 ppm concentration of *B. bassiana* on the mean malformed pupae (0.67 ± 0.047) was not significantly differed (3.59). A mutated crisis in the adult emergence was observed in *I. fumosorosea* fractions treated *S. litura* which showed 88 % adult emergence over *B. bassiana* and *P. variotii* with respect to 80 and 66 % (Fig. 1). However the performance of fraction on fecundity and hatchability of *S. litura* was immense. In addition to that, they shortened and mutated the each stage up to adult emergence in a panic and adverse way. Hence the fractions were not only pathogenic but also mutagenic to the *S. litura*. However, the instars treated with selected EPF showed significant difference in the treatment with respective fractions of EPF.

**Table 5:** Effect of fraction of entomopathogenic fungi on the fecundity and hatchability of against *Spodoptera litura*

Fungi	Concentration (ppm)	Eggs laid/batch	Eggs hatched	Pupa emerged	Malformed pupa	Adult emerged
*Isaria fumosorosea*	Control	176.04 ± 0.049^a^	95.45 ± 0.106^a^	96.00 ± 0.047^a^	0.00^a^	92.00 ± 0.433^a^
	1	167.97 ± 0.055^b^	94.61 ± 0.120^b^	76.00 ± 0.064^b^	4.00 ± 0.047^b^	72.00 ± 0.047^b^
	2	124.33 ± 0.085^c^	87.09 ± 0.180^c^	72.00 ± 0.623^c^	4.00 ± 0.117^c^	68.00 ± 0.004^c^
	3	84.03 ± 0.057^d^	42.00 ± 0.047^d^	28.00 ± 0.458^d^	12.00 ± 0.108^d^	16.00 ± 0.816^d^
	4	67.03 ± 0.057^e^	40.29 ± 0.064^e^	24.00 ± 0.204^e^	12.00 ± 0.131^e^	12.00 ± 0.816^e^
	CD at 0.01	0.049	0.028	0.079	0.353	0.101
	CD at 0.05	0.034	0.019	0.056	0.248	0.071
*Paecilomyces variotii*	Control	148 ± 0.020^a^	98.46 ± 0.080^a^	98.93 ± 0.016^a^	0.33 ± 0.471^c^	99.91 ± 0.115^a^
	1	118 ± 0.612^b^	89.78 ± 0.128^b^	84.00 ± 0.081^b^	0.67 ± 0.471^c^	84.00 ± 0.084^b^
	2	114 ± 0.122^c^	72.88 ± 0.080^c^	80.00 ± 0.151^c^	4.00 ± 0.365^b^	76.00 ± 0.426^c^
	3	96 ± 0.188^d^	55.76 ± 0.166^d^	68.00 ± 0.147^d^	4.00 ± 1.247^b^	60.00 ± 0.204^d^
	4	87 ± 0.069^e^	51.04 ± 0.376^e^	52.00 ± 0.440^e^	8.00 ± 0.081^a^	44.00 ± 0.382^e^
	CD at 0.01	0.263	0.459	3.906	3.592	3.526
	CD at 0.05	0.143	0.322	2.746	2.526	2.479
*Beauveria bassiana*	Control	149 ± 0.131^a^	92.61 ± 0.201^a^	99.91 ± 0.115^a^	4.00 ± 0.540^c^	96.00 ± 0.358^a^
	1	136 ± 0.099^b^	86.76 ± 0.147^b^	92.00 ± 0.092^b^	4.00 ± 0.071^c^	88.00 ± 0.128^b^
	2	118 ± 0.184^c^	76.27 ± 0.257^c^	84.00 ± 0.154^c^	4.00 ± 0.094^c^	80.00 ± 0.154^c^
	3	96 ± 0.062^d^	56.25 ± 0.277^d^	48.00 ± 0.540^d^	18.00 ± 0.108^a^	40.00 ± 0.104^d^
	4	84 ± 0.201^e^	32.54 ± 0.148^e^	32.00 ± 0.071^e^	12.00 ± 0.530^b^	20.00 ± 0.157^e^
	CD at 0.01	0.331	0.529	0.484	0.391	0.786
	CD at 0.05	0.200	0.319	0.292	0.235	0.474

Values are mean ± SD of three replication

^a,b,c,d^ *CD* critical difference values were significant at 0.01 and 0.05 % level

## Discussion

In this study, the tested fractions showed good sign of infection during the life cycle of *S. litura*. Vey et al. (2001) reported the in vivo effects of fungal metabolites in insects, measured as growth depression and changes in mortality, fertility, egg viability and metamorphosis. In the present study, a drastic decline in food consumption was observed with fraction of *I. fumosorosea* compared to *B. bassiana* and *P. variotii*. It was well supported by Tefera and Pringle (2003) who observed that, the significant reduction in consumption has been attributed to the production of toxic substances by the entomopathogenic fungi inside the host that lead to mechanical disruption in the insect structural integrity. The drastic reduction in food consumption in the present study was supported by Assaf et al. (2005) who stated that the reduction in feeding associated with the production of toxins by the fungus *I. fumosorosea.*

Sahayaraj and Tomson (2010) observed 33.34 % reduction in bodyweight of *Dysdercus cingulatus* treated with metabolites of *B. bassiana* fraction 2 (BBF2). It supported the present study that, the reduction in the range of 0.02–0.010 mg in larval weight of *S. litura*. Similar observation was also attained by Malarvannan et al. (2008) who found reduction in the larval weight of *S. litura* treated with the fractions of *Argemone mexicana* during development and observed the formation of shriveled pupa.

The early instar larvae of *S. litura* had shown normal digestion compared to control while proceeding the larvae failed to show a good sign of digestion process in terms of reduced AD. The higher AD values in the early instars of infected larvae may be due to the little consumption of the treated part. Likewise, the lower AD values in the late instars were because these caterpillars consumed food indiscriminately to meet the demand for energy and nitrogen (Hussain et al. 2009). In support of the present investigation, Hussain et al. (2009) observed higher AD values in *Ocinara varians* larvae infected with entomopathogenic fungi compared to the healthy control. The present investigation recorded reduced conversion potential (digested as well ingested food) of the treated larvae. Slansky and Scriber (1982) found that the utilization efficiencies (ECI) of 11 predaceous insects were between 4 and 75 %, while in the present investigation, the isolate *I. fumosorosea* fraction treated larvae had shown comparatively higher reduction in ECD and ECI which ranged from 75 to 46 % with respect to III to V instar larvae. In support of the present study, Sintim et al. (2009) observed 45.8 % ECI value in *S. litura* when fed with artificial diet. Fraenkel (1981) states that, under suitable conditions, a growing insect could convert maximum of 2/3 of its ingested food to body materials remaining will be utilized for metabolic processes.

Abnormal reduction in hatchability of eggs in *I. fumosorosea* treatment was in accompany with Leckie et al. (2008) observed delayed development, lower weights and high mortality of larvae of *Heliothis zea* when fed on diets containing mycelia of *B.* bassiana. Malarvannan et al. (2010) observed the complete arrest of fecundity by 2.4 × 10^7^ spore mL^−1^ concentration of *B.* bassiana. Similarly, Gindin et al. (2006) reported reduction of 80–82 % in the hatchability of red palm weevil adults, *Rhynchophorus ferrugineus* treated with *B. bassiana.* From this it was confirmed that, the deleterious impact of this insecticidal toxin or fraction on the larval anatomy has been noticed.

## Conclusions

Surveillance of impact of fungal fraction on growth and development of *S. litura* was performed in the present study. Sub lethal dose of *I. fumosorosea* fraction had significant impact on the consumption and digestion rate of *S. litura.* There was severe damage in the midgut regions were noticed. In addition to that, the next generation seeds, the eggs, were heavily suffocated in development and thereby *I. fumosorosea* fraction severely reduced the hatching and thus leads to significant production of malformed pupae for the next generation. Therefore, *I. fumosorosea* fraction could be reliable biocontrol agent that can highly recommend for *S. litura* management in cotton as well as sunflower field.

## Methods

### Isolation of entomopathogenic fungi

One gram of soil was diluted with 10 ml of distilled water and was serially diluted. From each dilution, 100 µL was plated on Potato Dextrose Agar (PDA) medium and it was fortified with streptomycin (10 mg/100 ml). It was allowed to grow for 7 days at 27 ± 2 °C in the respective media. After 7 days of incubation, the fungal colony was identified and was sub-cultured in Sabaroud Dextrose Agar (SDA). In the case of cadavers, each cadaver is carefully held with a light forceps and the conidia drawn slowly into a vial using a 00 camlin brush. Five milli-gram of dry spore was taken and added with 10 ml of sterile 0.02 % tween 80 solution. The sterilized SDA medium was transferred into sterile petridishes and test tubes that were then inoculated with pure conidia of entomopathogenic fungi by streak plate method (Haraprasad et al. 2001) The isolated fungi were transferred on to Potato Dextrose Agar (Hi-Media, India) petri dishes (9 cm in diameter) (Borosil^®^) and incubated at 25 °C for 1–2 week to produce conidia which is used as inoculums for the further study.

### Screening of secondary metabolites

After 2 weeks of incubation, dense sporulated PDA plate was used for harvesting the inoculum preparation. The plate was flooded with 20 mL of sterile distilled water supplemented with 0.02 % tween 80 (Hi-Media, India) and scraped with stainless steel spatula (Hi –Media, India). It was then filtered through muslin cloth and the resulted spore solution was subjected for spore count using Haemocytometer. The spore concentration was then adjusted to 1 × 10^8^ and 1 mL from this stock was poured into the Potato dextrose broth (PDB) (Hi-Media, India) for secondary metabolite production. After 14 days of incubation, crude extracts of the cultured broth were obtained following the method reported by Hu et al. (2007) with minor modification. The thick mycelia mat was removed and culture medium was harvested and centrifuged (Remi, India) at 8000 rpm for 10 min. The supernatant was extracted with ethyl acetate (Sample: Ethyl acetate = 5:2, v/v) and the organic phase was evaporated by placing it in incubator at 40 °C. The concentrate was diluted with 4 times volume of water and incubated at 4 °C overnight until the precipitation was observed. Finally, dried precipitate was used for bioassay as secondary metabolite. The precipitate was dissolved in ethyl acetate and used for feeding experiment.

### Feeding experiment

The first instar larva of *S. litura* was separated from stock culture and fed with *Ricinus communis* leaves ad libitum. After the larvae reached the III instar, the initial weight of the larvae and leaves provided to them were calculated using digital balance. The leaf consumption rate was calculated from III to V instar along with the growth rate of the larvae. The faecal matter of these larvae was also collected at each stages were dried and weighed from which the a *P. variotii* milation rate of the larvae was calculated. It was adopted to larvae of *S. litura* to calculate its consumption index (CI), growth rate (GR), approximate digestibility (AD), efficiency of conversion of digested food (ECD) and efficiency of conversion of ingested food (ECI) by gravimetric analysis (Waldbauer 1968) by using the following formula.

### Consumption index (CI)

Consumption index (CI) or the rate of feeding relative to the weight of the insect in a definite time can be expressed as:

$${\text{CI}} = \frac{\text{F}}{\text{TA}}$$

where, F is the weight of food eaten; A is the mean weight of insect during the feeding period, T is the duration of the feeding period (Days)

### Growth rate (GR)

This measurement explains the rate at which the digested matter is available to the insect during the experimental period.

$${\text{GR = }}\frac{\text{Weight gained by the insect}}{{{\text{Duration of feeding period }}\left( {\text{Days}} \right) \times \left( {\text{Mean weight of insect during the feeding period}} \right)}}$$

### Approximate digestibility (AD)

$${\text{AD}} = \frac{{{\text{Weight of food ingested}} - {\text{weight of faeces}}}}{\text{Weight of food ingested}} \times 100$$

Earlier workers have used to call approximate digestibility as “co-efficient of utilization”, “coefficient of digestibility” and “degree of absorption” for express *P. variotii*ng the digestibility of food material. Waldbauer (1968) pointed out that this measure is misleading and should be referred to as “approximate digestibility”.

### Efficiency of conversion of digested food (ECD)

$${\text{ECD = }}\frac{\text{Weight gained by the insect}}{\text{Weight of food digested}} \times 100$$

The amount of food digested can be calculated by subtracting the weight of faeces from the weight of food ingested. This index has also been termed by some workers as “coefficient of growth”.

### Efficiency of conversion of ingested food (ECI)

Efficiency of conversion of ingested food measurement indicate the overall efficiency of the insect to utilize the food for growth.

$${\text{ECI}} = \frac{\text{Weight gained by the insect}}{\text{Weight of food ingested}} \times 100$$

### Statistical analysis

Data of fecundity and hatchability both control and treatment was subjected to one tailed t test by using Statistical Packages for Social Sciences version 17. The critical difference (CD) value was calculated by using the software WASP 1.0.
